# The growing need for resources to help older adults manage their financial and healthcare choices

**DOI:** 10.1186/s12877-017-0477-5

**Published:** 2017-04-11

**Authors:** Stephanie MacLeod, Shirley Musich, Kevin Hawkins, Douglas G. Armstrong

**Affiliations:** 1 Advanced Analytics, Optum, 315 E. Eisenhower Parkway, Suite 305, Ann Arbor, MI 48108 USA; 2 AARP Services, Inc., 650 F. Street, N.W, Washington, D.C., 20004 USA

**Keywords:** Financial health literacy, Financial literacy, Health literacy, Financial health, Older adults, Medicare, Retirement planning

## Abstract

**Background:**

Both financial literacy (managing personal finances) and health literacy (managing personal health) become increasingly important for older adults, potentially impacting their quality of life. Resources in these constructs of literacy tend to be distinct, although the skills and decision-making involved overlap as financial issues impact healthcare choices. Thus the primary purpose of this commentary is to propose a new area of research focus that defines the intersection of financial and health literacy (i.e., financial health literacy).

**Methods:**

We conducted a limited literature review related to financial, health, and health insurance literacy to demonstrate gaps in the literature and support our position. Online search engines were utilized to identify research in our primary areas of interest.

**Results:**

We define the intersection of financial and health literacy as an area of need labeled financial health literacy, with a focus on four domains. These include: 1) the ability to manage healthcare expenses; 2) pay medical bills; 3) determine health needs and understand treatment options; and 4) make sound healthcare decisions with financial resources available. Despite some overlap with health insurance literacy, financial health literacy would define an area of need encompassing health management choices and health plan selections integrated with other financial management issues including living arrangements, financial planning, and retirement planning.

**Conclusions:**

Potential initiatives should be considered to help at-risk older adults find resources to improve their financial health literacy, which in turn will enhance their abilities to manage medical choices in the environment of an increasingly complex healthcare system.

## Background

In the US, over 46 million Americans are age 65 or older, a number that is increasing rapidly. This population is projected to nearly double by 2050 [1–3] making them the fastest growing group in the US, dominated by the Baby Boomers (born from 1946 to 1964), who are reaching retirement and Medicare eligibility. Thus efforts to help older adults age successfully with optimal health outcomes, access to quality care, financial stability, and tools to make decisions independently have become a priority. Both financial and health literacy become increasingly important with age, impacting older adults and their quality of life [4, 5]. As older adults retire, they face increased responsibilities for managing both financial and healthcare needs, assuming decision-making roles previously managed by employers with employer-based medical, drug, and life insurance plans and, perhaps more importantly, associated supporting resources. Meanwhile, in a rapidly evolving healthcare environment, healthcare treatment choices and health plan structures are increasingly complex, out-of-pocket cost-sharing is higher, and prescription drug coverage is restricted by Medicare Prescription Drug (Part D) plans. In addition, many older adults demonstrate low levels of financial and health literacy to deal with these multi-faceted issues [6–12].

Financial literacy encompasses the ability to understand financial concepts and make sound financial decisions, primarily regarding housing, investments, and long-term savings [5, 13–16]. Meanwhile, health literacy encompasses the ability to understand and process health information to make the best personal choices regarding the utilization of healthcare services, treatment planning, and end-of-life healthcare planning [17–19]. A separate but related area of focus, health insurance literacy describes an individual’s knowledge and ability to find and evaluate information specific to health plans, select the best plans, and use the plans appropriately [20–22]. In this commentary, we are proposing a previously undefined area of literacy, financial health literacy (FHL). While FHL overlaps with some areas of health insurance literacy (such as choosing and using advantageous medical plans), it would also encompass understanding the financial dimensions of treatment options integrated with other life choices and financial planning to maximize quality of life as one ages. As we will show, to date, the overlap of closely intertwined financial and healthcare decisions has not been recognized in the scientific literature.

## Statement of purpose

**Fig. 1:**
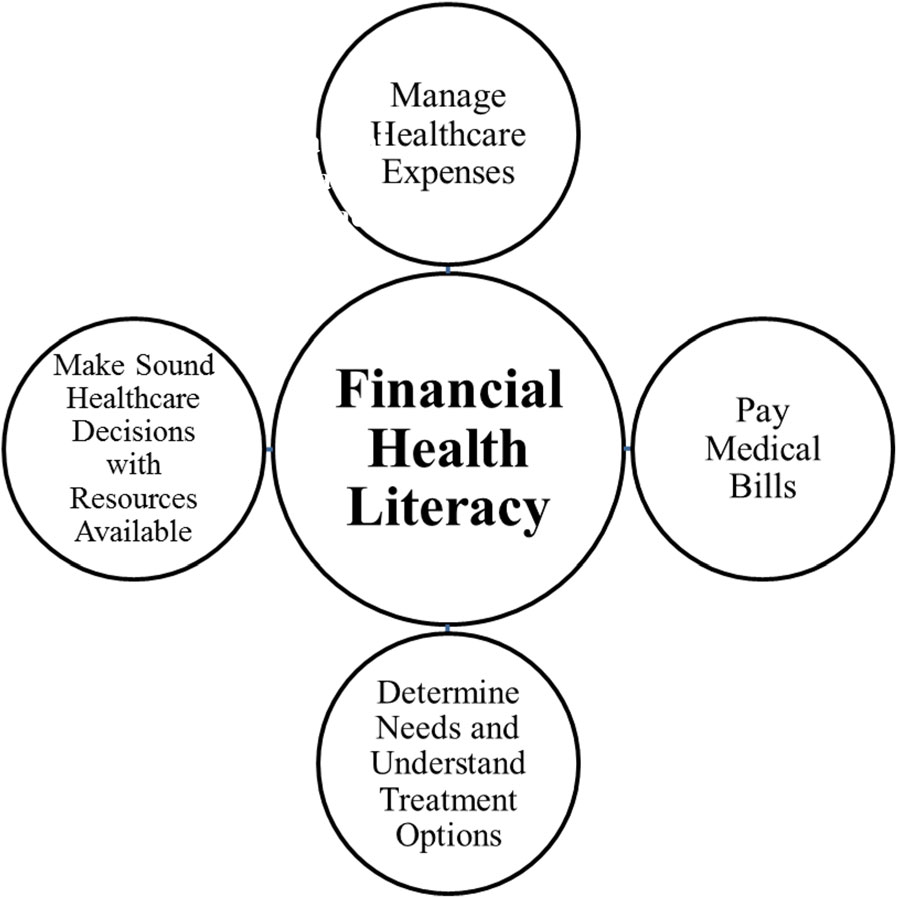
The Key Domains of Financial Health Literacy

A gap in the literature exists regarding the significant overlap of financial literacy and health literacy, as well as its critical importance to older adults. Thus the primary purpose of this commentary is to propose a new area of focus to better define this intersection as financial health literacy (Figure 1): the capacity to understand and apply financial information to plan for healthcare expenses and to make sound healthcare and treatment choices while managing other household finances effectively. In doing so, we will describe a limited overview of existing research in relevant areas of financial, health, and health insurance literacy to demonstrate gaps in the current literature and support our position on the need for establishing resources to address this newly defined area. Additionally, we will outline our proposed key domains of FHL (Table 1), and thereby suggest potential conceptual skills in order to identify those at risk for low FHL, especially later in life.

**Table 1:** Potential Domains of Financial Health Literacy and Related Conceptual Skills

Domains of FHL	Conceptual Skills
Manage Healthcare Expenses	Budget effectively for monthly healthcare expenses
	Manage healthcare and household bills simultaneously
Pay Medical Bills	Understand and pay medical bills, household bills, and other living expenses
Determine Health Needs and Understand Treatment Options	Determine amount of savings and income needed for monthly expenses, including health care and other expenses
	Understand medical care options
	Determine estimated long-term healthcare expenses
	Understand premiums, deductibles, copayments, and coinsurance
	Determine prescription drug coverage and other insurance needs
	Understand the implications of end-of-life care options
Make Sound Healthcare Decisions with Financial Resources Available	Independently choose appropriate Medicare, Supplement, and drug plans as needed
	Change insurance plans as needed
	Independently choose appropriate treatment and medical care options
	Plan for end-of-life care choices

Note: Each conceptual skill in Column 2 would potentially generate a question or set of questions for a scale for FHL

## Methods

To inform this commentary and support our position, we conducted a thorough yet limited review of the scientific literature relevant to our purpose in the areas of financial, health, and health insurance literacy. We first searched for “*financial health literacy*” and reviewed the results, which provided no existing research studies specifically dedicated to the domains of FHL as we propose to define it. Therefore we gathered and summarized the most relevant literature on the related but separate areas of literacy discussed here (financial, health, and health insurance literacy).

Online search engines, primarily PubMed, Medline, Google Scholar, and a mainstream Google search, as well as references from relevant articles, were utilized to identify research in our areas of interest. Across these sources, we used several primary search terms, including: *financial literacy, financial literacy measures/scales, financial literacy resources older adults/seniors, financial planning, health literacy, health literacy measures/scales, health literacy resources older adults/seniors, health insurance literacy, health insurance literacy measures/scales, health insurance literacy resources older adults/seniors, older adults/seniors, Medicare, retirement savings,* and *retirement planning*. Each search term returned a number of results too large to review individually. Thus we narrowed our results to limit the search for these exact phrases to better identify research pertaining directly to older adults. Additionally, we focused on publications dated 2006 or later as these would be most relevant to the current healthcare system and Medicare Part D, although earlier research papers with important background information or definitions were considered. Finally, publications specifically related to older adults’ health or medical decisions based on their treatment options (with no consideration for costs) or other health factors alone were excluded, as they were not relevant to our objective of examining the overlapping roles of financial and health literacy. Research studies describing the reasoning for, or the process of, making medical decisions due to a lack of money could have been relevant to this commentary; however, we did not identify specific studies in this area to include. Thus our commentary is based on the publications deemed most relevant to our purpose.

## Results of search

For brevity, results are summarized for only the primary search terms used to gather research. *Financial literacy* returned approximately 1700 results; *health literacy* returned over 10,000 results; and *health insurance literacy* returned 390 results, most of which overlapped with general health literacy and were not specific to health insurance literacy. Furthermore, many results for both *financial literacy* and *health literacy* overlapped with general literacy topics or areas not useful for this commentary. Thus the following numbers of publications were selected to review in detail: *financial literacy: 59; health literacy: 11; health insurance literacy: 10.*

After further review of the most relevant publications, the final number ultimately cited in this commentary totals 43, with 42 articles published in 2006 or later, and the remaining published earlier. The resulting commentary is based on this limited literature review, which was sufficient to support our position on FHL as a unique area of need.

## Definitions

### Financial literacy

Financial literacy has been defined with validated measures in the scientific literature, with several variations: the ability to understand financial information to make informed decisions [13]; to perform simple calculations and know basic fundamental financial concepts needed to make effective financial decisions [14]; to manage money and finances in ways consistent with one’s self-interest [15, 16]; and to understand and act on information from diverse sources for optimal financial functioning [5]. Specific financial decisions include savings calculation, retirement planning, Social Security benefit choices, end-of-life financial planning, living arrangements, wealth distribution, and life insurance. Financial expertise tends to focus on financial planning prior to and during retirement but not specifically on managing healthcare decisions and expenditure ramifications later in life. Research indicates that older adults may lack basic financial knowledge [13] to manage their money successfully while living on fixed incomes. In research studies with older adults (age 50 and older), the reported prevalence of adequate financial literacy based on existing measurement scales ranges from about 18% [10] to about 57% [14, 17]. Financial literacy appears to decrease with increasing age [16].

### Health literacy

Health literacy has been defined with validated measures as the capacity to obtain, communicate, process, and understand basic health information and services to make appropriate health decisions [17–19]. Health literacy generally focuses on managing health needs rather than how to coordinate those needs with healthcare treatment and payment options, including both medical and drug plans. As with financial literacy, older age and lower education and income have been associated with low health literacy. Research indicates that among all American adults (age 16 and older), 36% are considered to have low (“basic” or “below basic”) health literacy [4, 5, 23, 24]. The US Department of Education’s National Assessment of Adult Literacy (NAAL) found that low health literacy is especially prevalent among the elderly: 59% of adults age 65 years and older scored in the lowest two ranges of health literacy (“basic” and “below basic”) [23, 24]. In addition, according to the National Network of Libraries of Medicine (NNLM), 68% of older adults have difficulty understanding numbers and calculations; 71% have difficulty using print materials; and 80% have difficulty using health-related forms, charts, and other documents [9, 12].

### Health insurance literacy

Compared to financial and health literacy, health insurance literacy is an emerging area of focus, with fewer measurement scales in use or research studies published [21, 22]. In fact, among over 200 general health literacy studies in the literature, none have specifically examined the ability to use health insurance plans [9, 22].Health insurance literacy is defined as, “the degree to which individuals have the knowledge, ability, and confidence to find and evaluate information about health plans, select the best plan for their own (or family’s) financial and health circumstances, and use the plan once enrolled” [21, 22]. Research suggests many Americans have low health insurance literacy: for example, 88% of US adults cannot calculate their share of health insurance costs [9, 22]. Despite some overlap with aspects of health insurance literacy, FHL would be a unique area of need encompassing broader financial considerations and conceptual skills other than those related specifically to insurance plan selection. The additional suggested domains and conceptual skills of FHL are show in Table 1.

### Financial health literacy

Research indicates that financial, health, and health insurance literacy are closely intertwined and especially impactful in terms of later-life decision-making [5, 25]. Among older adults, low financial and health literacy are correlated; both are associated with similar characteristics and outcomes [5, 26]. However, this intersection labeled FHL has yet to be recognized as a standalone area of need or to have defined resources that might support older adult health and financial decision-making. We would propose to define FHL more specifically as the capacity to understand and apply financial information and available financial resources to make sound healthcare and treatment choices while also managing other household expenses effectively. FHL, as we envision this overlap, would encompass four key domains: 1) the ability to manage healthcare expenses; 2) pay medical bills; 3) determine health needs and understand treatment options; and 4) make sound healthcare decisions with the financial resources available (Table 1 and Figure 1). We derived these proposed key domains based on similar skills that would be required for proficiency in the established overlapping constructs of financial literacy and health literacy as described extensively in the literature [9, 12–19]. Defining and establishing FHL as a separate area of need with these key domains would be a necessary first step in developing a scale to determine its impact among older adults and subsequent supporting resources.

## Measurement scales

### Financial literacy

Currently, there is no single screening question to measure basic financial literacy, but longer validated measures exist. Several commonly used surveys, primarily the University of Michigan Health and Retirement Study (HRS) [27] and the RAND American Life Panel (ALP) [28], have incorporated three items developed specifically to address financial literacy [9, 16]. These items are based on the four key components of financial capability, including short-term money management, future planning, managing financial products/services, and numeracy [9, 16, 26–28], all of which have been established as important skills for older adults’ financial stability. However, the actual questions included in these surveys tend to focus more on very specific financial calculations or complex investing strategies rather than general concepts older adults need to understand in order to manage finances. To be relevant, these concepts should focus more specifically on, for instance, saving enough money for retirement and potential medical expenditures, planning for other unforeseen expenses or emergency savings, budgeting on a fixed income, and balancing living expenses including medical and drug insurance premiums. In addition, the items do not specifically address the key concerns of older adults when trying to manage their finances and pay for later-life expenses. For example, the first item of those used in the HRS and ALP, with five answer choices provided, reads: *“Suppose you had $100 in a savings account and the interest rate was 2% per year. After 5 years, how much do you think you would have in the account if you left the money to grow?”* The second item, also with five responses, reads: *“Imagine that the interest rate on your savings account was 1% per year and inflation was 2% per year. After 1 year, how much would you be able to buy with the money in this account?”* Finally, the third item is as follows: *“Please tell me whether this statement is true or false. Buying a single company’s stock usually provides a safer return than a stock mutual fund.”* Among Americans over age 50, only half answered the first two items correctly; only one-third answered all three items correctly [9, 16]. In fact, only 44% of those with a college degree could answer all three items correctly.

Other measures of financial literacy include the Financial Industry Regulatory Authority (FINRA) National Financial Capability Survey [7], which links indicators of financial capability to demographic and other characteristics. FINRA’s Susceptibility to Scams Scale assesses financial decision-making skills and susceptibility to financial victimization [29]. Another measure, the Lichtenberg Financial Decision-Making Rating Scale (LFDRS), uses 77 questions designed to assess financial situation awareness, decision-making ability, and exploitation vulnerability; it has been validated with older adults [15, 30, 31]. Elsewhere, the Independent Living Scales and Managing Money Subscale (ILS) consists of 68 items designed to evaluate functional ability among adults age 65 and older [32]. The ILS primarily measures memory, orientation, and the ability to manage home matters and finances [31, 32]. Generally, however, these measures focus on cost of living expenses rather than medical expenses, and do not include all of the conceptual skills important for FHL.

The common chosen resource for financial literacy would most likely be a financial planner specializing in later-life financial planning and retirement needs. Existing money management resources designed for older adults tend to focus on basic financial concepts, such as investment planning to cover living arrangements and expenses in retirement as well as Social Security options. Websites designed for this purpose often feature financial tools related to economic security, debt management, scams, and tips for aging workers [33]. Larger financial institutions also offer financial planning assistance, such as USAA’s online Financial Readiness Score tool (www.usaa.com) that allows members to assess their preparedness for retirement and offers access to financial advisors by phone [34]. As another example, AARP’s website (www.aarp.org) features several financial offerings including free tax preparation, fraud and scam alerts, and other tools [35]. These resources, however, focus on general finances and savings prior to and during retirement rather than the complex issues related to planning for health plan premiums, healthcare expenses, and the large savings needed specifically for late-life expenditures. In addition, locating disparate financial and healthcare planning resources may be as time-consuming and difficult for older adults as understanding and utilizing the information to meet their individual needs.

### Health literacy

Compared with financial literacy, health literacy research has been developed more extensively. Notably, in our review of the literature on these topics, a search for *financial literacy* returned approximately 1700 results while *health literacy* returned over 10,000 results, indicating a greater research base. Furthermore, measurement of health literacy across various settings and populations is commonly based on one widely used and accepted validated screening item: *“How confident are you filling out medical forms by yourself?”* [36, 37]. Using this screening item, researchers have reported a high prevalence of both limited and marginal health literacy among adults, including seniors [37]. While this single item is not truly considered a “gold standard,” it does allow for identifying individuals who may be at risk for low health literacy, much like the purpose a screener for FHL could serve.

Research on literacy assessment suggests the importance of measuring the impact of both objective (actual) and subjective (perceived) knowledge in an individual’s decision-making process [38]. The single screening question commonly used to assess basic health literacy, as described previously, is essentially subjective, yet various longer validated measures do include objective measurement items. One example is the Consumer Assessment of Healthcare Providers and Systems (CAHPS) [39] Clinician and Group Surveys: Supplemental Items for Adult Survey 2.0 [41], which includes 31 items on health literacy. Another measure, the Rapid Estimate of Adult Literacy in Medicine, Revised (REALM-R), helps medical professionals identify patients with low health literacy by assessing their ability to read and understand common medical terms [40]. Elsewhere, the University of Michigan’s HRS includes nine questions specific to health literacy [27]. However, as with the financial literacy scales, some items used in these health literacy measures are not easily adaptable to all general older populations as they incorporate concepts that may be unfamiliar to some older individuals. For example, Michigan’s HRS asks, *“How many years is it recommended that the average person who has had a normal colonoscopy should wait before having another colonoscopy?”* Although an objective question, this item may not reflect common knowledge, as the frequency of recommended colonoscopies varies depending on test results. Furthermore, another item in this section of the HRS appears to be more opinion-based, and thus responses may be an inaccurate representation of health literacy: *“Do you think regular colon cancer screening for people over age 50 does or does not reduce the risk of dying from colon cancer?”*

Various health-related resources provide general information about common concerns and condition management. For older adults, a general practitioner or other physician is likely to be a common chosen source of information. Older adults may trust their physicians to provide the best advice on their health needs, although physicians may not address medical costs or insurance billing with their patients. Multiple websites, such as those of the Centers for Disease Control and Prevention (CDC), Mayo Clinic, and WebMD, do provide guidance on medical conditions; some offer an online symptom checker or search feature to match symptoms with certain conditions. For older adults seeking in-person assistance, local health fairs or senior centers may provide access to nurses or other providers to discuss health concerns. Free healthcare services such as weight measurement and blood pressure, cholesterol, and diabetes testing are often available as well. Other personalized resources include pharmacists at retail pharmacy or drugstore outlets. Many older adults who take prescription medications likely know where to go for this advice, such as a neighborhood pharmacy, and may even have a pharmacist they personally know and trust. Finally, mainstream media publications targeting older audiences often feature articles on health care, common conditions, healthy lifestyle strategies, and treatment/medication options. Compared to some of the financial resources, these health-related options may be more commonly known or easier to access. However, they tend to focus strictly on health issues rather than the expenses associated with managing health later in life.

### Health insurance literacy

While health insurance literacy has been defined, this area of literacy has not been measured extensively, and no widely used assessment scale exists [21, 22]. Health insurance literacy is described as a “subset” of health literacy, but existing health literacy measures do not effectively measure the skills and behaviors involved [22]. Among existing measures of health insurance literacy, none have been tested for both reliability and validity or were developed specifically to assess insurance purchasing behaviors [21]. Recently, the Health Insurance Literacy Measure (HILM) was developed as a self-assessment of individuals’ ability to select and use health insurance plans [21], but it has not been extensively used.

For health insurance literacy, a commonly used resource would likely be a representative with a specific insurance plan or an independent broker to assist with health plan choices. These individuals can help consumers to select healthcare plans or medical care options and provide information on healthcare expenses. Other existing resources geared toward evaluating insurance options and planning for healthcare expenses could serve as examples to inform further development of specific tools to support the additional domains of FHL. For instance, online resources have emerged as a common service offered by various entities. AARP’s Benefits Quick Link, for one, helps members determine whether they qualify to save money on health care, and an online long-term care calculator provides guidance in planning for long-term care expenses, which would be an important component of FHL [41]. Elsewhere, government websites and larger retail pharmacy/drugstore chains offer interactive drug plan comparison tools and/or access to health plan advisors [41].

As an alternative to online resources, older adults may seek assistance on their own from well-known brands, such as AARP or BlueCross BlueShield (BCBS), as they have highly visible products and readily available contact information. These resources could potentially assist those who need help using their limited finances most effectively. However, additional, easily accessible options in this area are still needed to improve and support FHL among older adults.

### Financial health literacy

Currently, no measurement scales exist to assess the conceptual skills involved in FHL. An initial screening question for FHL, similar to that for health literacy, could be useful; for instance: *“How confident are you with managing healthcare expenses independently, including paying bills?”* Further development of a longer measure would also be instrumental in establishing FHL and identifying those at risk for low FHL. The key overarching domains should first be established (Table 1), each encompassing several conceptual skills to consider in developing a measure. As Table 1 illustrates, FHL would incorporate health insurance literacy; however, FHL would also include three additional domains.

For FHL, further development of needed resources in this area could include a centralized website combining links to various tools and information targeting older adults, which could be simple to find and explore. A website could also include contact information or phone/email access to personal financial health advisors who specialize in the intersection of financial management, healthcare treatment choices, living expenses, and insurance issues. If marketed and advertised effectively, these resources could provide one source of guidance for older adults in planning their expenses and finances without the need to search in different places for information across all of these critical areas. Even those with limited internet experience could likely search and explore links presented in one space. In addition, many websites designed to provide this type of information also offer assistance by phone, such as www.usaa.com, which includes a toll-free phone number for access to financial advisors available for advice and questions on overlapping financial and health issues. Other existing websites designed to assist older adults include the AARP member website, available at http://member.aarp.org. This website provides various health, financial, fraud protection, and safety-related resources for older adults and those involved with their decision-making or care.

Another option with potential promise may be services and advisors providing unbiased assistance with managing healthcare expenses and/or making medical plan and treatment choices. This type of offering could be comparable to resources available through the National Association of Area Agencies on Aging, which provides educational programs in local areas throughout the US as well as online (http://www.n4a.org/), or through other organizations such as local United Way offices. Resources would need to be user friendly, accommodate all reading levels, and include enough detail to be useful in planning. In addition, those without regular computer access or computer literacy would likely need alternative options, such as services accessible over the phone or in person, brochures, handouts, or other written materials, or individual sessions and classes covering aspects of FHL. For instance, local senior centers could potentially serve an important role in this area by providing accessible, useful, and cost-free offerings, and these centers would likely be well known to seniors and their families. Senior centers could also offer free or low-cost access to professionals specializing in these areas who would provide individualized advice or assistance in managing overlapping late-life expenses. In addition, organizations such as USAA (http://www.usaa.com) offer retirement planning and financial advice, often along with healthcare planning resources.

Finally, those seeking help, including not only older adults but also their family members, adult children, and caregivers who help to make decisions, would need to know how to find these resources in order to take advantage of them. Thus future directions in this area would ideally include an aspect of communication or targeted marketing to promote resources for older adults and those who have a role in influencing their decision-making process. This responsibility could, in part, fall to healthcare providers or others within the finance and healthcare industries who interact with older individuals.

## Discussion

Research demonstrates that many older adults have low financial, health, and health insurance literacy, each of which become increasingly important due to the decisions they face, especially with declining health and fixed incomes. These decisions were likely previously made by employers who offered employer-based medical, drug, life, dental, and vision insurance products and associated resources to help in making choices that best fit the given employee’s circumstances. Older adults have no parallel resource for getting advice in managing these areas of health and finances. Older adults tend to consume more healthcare services and have higher healthcare expenditures than younger groups, thus financial and health management skills become imperative.

As older adults face critical decisions, including savings needed, retirement plans, Social Security benefit choices, end-of-life plans, life insurance, Medicare, Medicare Supplement and Prescription Drug (Part D) plan selections, and important medical care decisions, resources to support and assist with decision-making become increasingly necessary. Recent estimates suggest that a 65-year-old couple should plan to have about $250,000 saved strictly to cover medical expenses post retirement [42, 43]. In addition, money management to pay for monthly premiums, out-of-pocket copayments, unpredictable healthcare expenditures, long-term care expenses, and all other living expenses add to financial stress.

Establishing FHL would require development of a measurement scale to assess the conceptual skills involved, perhaps initially based on one screening question. As we have suggested, a screener for FHL, perhaps *“How confident are you with managing healthcare expenses independently, including paying bills?”* could also be useful in developing a longer comprehensive measure. Based on our proposed definition, the key domains of FHL would encompass the conceptual skills listed in Table 1. Each conceptual skill would generate specific questions to use in development of an assessment scale to identify older adults most at risk for low FHL. It is reasonable to suggest that without these conceptual skills, an individual would also struggle to manage not only healthcare but also living expenses; pay bills for all of these expenses simultaneously; determine their needs and understand available options; and make sound decisions, not only about insurance plans but also medical care, treatment, and end-of-life options. A full-length measure assessing these skills could help to determine an individual’s level of FHL and, most importantly, identify at-risk older adults who would benefit from tools to enhance their literacy in this area.

## Conclusions

Defining FHL as a unique area of need may be the first step in simplifying the decision-making process for older adults as they address the burdens of managing healthcare expenses, other living expenses, and life-changing decisions. In this commentary, we have proposed a definition of FHL, an area of need encompassing four main domains: the ability to manage healthcare expenses, pay medical bills, determine needs and understand treatment options, and make sound healthcare decisions with the financial resources available. Although FHL would overlap with certain areas of health insurance literacy, it would involve other unique factors, such as the ability to plan for and pay for all late-life expenses and make affordable treatment choices with limited resources. In addition, we have suggested the conceptual skills within each domain that would be important to assess with a validated measurement scale to identify those most at risk for low FHL. By establishing the need for FHL and subsequently identifying those with low FHL, resources can be better targeted to help older adults and their caregivers more successfully manage their healthcare needs, options, and other living expenses while making affordable and sound decisions.

## Abbreviations

ALP: American Life Panel
BCBS: BlueCross BlueShield
CAHPS: Consumer Assessment of Healthcare Providers and Systems
CDC: Centers for Disease Control and Prevention
FHL: Financial Health Literacy
FINRA: Financial Industry Regulatory Authority
HILM: Health Insurance Literacy Measure
HRS: Health and Retirement Study
ILS: Independent Living Scales and Managing Money Subscale
LFDRS: Lichtenberg Financial Decision-Making Rating Scale
NAAL: National Assessment of Adult Literacy
NNLM: National Network of Libraries of Medicine
REALM-R: Rapid Estimate of Adult Literacy in Medicine, Revised
